# Bioinformatic analysis of differentially expressed profiles of lncRNAs and miRNAs with their related ceRNA network in endometrial cancer

**DOI:** 10.1097/MD.0000000000032573

**Published:** 2023-01-20

**Authors:** Fengfan Li, Chunlei Zhou, Shuxuan Li, Jingyu Wang, Ming Li, Hong Mu

**Affiliations:** a The First Central Clinical College of Tianjin Medical University, Tianjin, China; b Department of Clinical Laboratory, Tianjin First Central Hospital, School of Medicine, Nankai University, Tianjin, China; c Department of Gynecology, Peking University Second Hospital, Beijing, China.

**Keywords:** ceRNA, EC, lncRNA, miRNA, TCGA

## Abstract

Increasing evidence suggests that long non-coding riboneucleic acids (lncRNAs), as competing endogenous RNA (ceRNA), play a key role in the initiation, invasion, and metastasis of cancer. As a new hypothesis, the lncRNA-micro RNA (miRNA)-messenger RNA (mRNA), ceRNA regulatory network has been successfully constructed in a variety of cancers. However, lncRNA, which plays a ceRNA function in endometrial cancer (EC), is still poorly understood. In this study, we downloaded EC expression profiling from The Cancer Genome Atlas database and used the R software “edgeR” package to analyze the differentially expressed genes between EC and normal endometrium samples. Then, differentially expressed (DE) lncRNAs, miRNAs and mRNAs were selected to construct a lncRNA-miRNA-mRNA prognosis-related regulatory network based on interaction information. The Gene Ontology and Kyoto Encyclopedia of Genes and Genomes pathway enrichment analysis were performed on the genes in the network to predict the potential underlying mechanisms and functions of lncRNAs in EC. Kaplan–Meier method and the log-rank test were used for survival analysis. Based on the “ceRNA hypothesis,” we constructed a co-expression network of mRNA and lncRNA genes mediated by miRNA in the process of tumor genesis. Furthermore, we successfully constructed a dysregulated lncRNA-associated ceRNA network containing 96 DElncRNAs, 27 DEmiRNAs, and 74 DEmRNAs. Through Kaplan–Meier curve analysis, we found that 9 lncRNAs, 3 miRNAs, and 12 mRNAs were significantly correlated with the overall survival rate of patients among all lncRNAs, miRNAs, and mRNAs involved in ceRNA (*P* < .05). Our research provides a new perspective for the interaction among lncRNAs, miRNAs, and mRNA and lays the foundation for further research on the mechanism of lncRNAs in the occurrence of EC.

## 1. Introduction

Endometrial cancer (EC) is one of the 3 major malignant tumors of the female genital tract. In 2021, statistical data showed that the mortality rate of EC in the US was second only to ovarian cancer.^[1]^ The incidence and mortality of EC in Chinese women is increasing, and victims are becoming younger over time.^[2]^ Although the level of medical diagnosis and treatment is quite developed at present, the exact pathogenesis of EC is not clear, and its treatment is very limited. Therefore, further exploration of the molecular mechanism of the occurrence and development of EC, and finding effective therapeutic targets are of great significance for the protection of women’s health.

Research into long non-coding RNAs (lncRNAs) has opened up a new way to explore the pathogenesis of EC recently. lncRNAs are a class of endogenous ribonucleic acid (RNA) molecules larger than 200 bp and lacking the ability to code for proteins. lncRNAs were initially considered to be the “noise” of genome transcription and a byproduct of RNA polymerase II transcription and thus thought to have no biological function. In recent years, with the continuous research and understanding of lncRNA, it has been found that lncRNAs play an important role in the normal development of individuals, and the occurrence, development, and prevention of diseases.^[3]^

Increasing evidence shows that the interaction between lncRNA, micro RNA (miRNA), and its downstream target genes are closely related to the occurrence and development of tumors. Based on this, the competing endogenous RNAs (ceRNAs) hypothesis was proposed and has been confirmed by a number of researches. The ceRNA hypothesis is a new mode of regulation of gene expression. It is known that miRNA combined with messenger RNA (mRNA) can cause gene silencing, and ceRNA can regulate gene expression by competing with miRNA, thereby affecting the function of the cell.^[4,5]^ miRNA is an important factor in post-transcriptional regulation, and its activity can be regulated by lncRNA acting via “sponge” adsorption, and this kind of lncRNA is also called ceRNA. Because ceRNA, lncRNA and miRNA competitively bind to mRNA, this regulates the protein level of the encoding genes and participates in the regulation of the biological behavior of the cells.^[6]^ In recent years, lncRNA-miRNA-mRNA ceRNA regulatory networks have been identified in gastric cancer, lung cancer, liver cancer, and ovarian cancer and have been confirmed by various studies.^[7–9]^ However, lncRNAs that perform ceRNA functions in EC are still poorly understood. Therefore, there is an urgent need to investigate lncRNA expression regulation networks based on the ceRNA hypothesis, which can provide a theoretical basis for the diagnosis and treatment of EC.

With the extensive use of gene expression profiling chip technology research, there has been a large volume of data released to public database platforms. The Cancer Genome Atlas (TCGA) program was originally developed by the National Cancer Institute and the National Institute of Human Genome Research, for which >30 human cancer types have been collected, and around 10,000 patients’ samples and clinicopathological information have been included; the data are all free and open. The ultimate goal of TCGA is to construct a complete set of “maps” of all cancer genomes. In the TCGA database, free data include not only comprehensive and detailed clinical case data, but also patient-level molecular sequencing results, and the integration of these databases can offer the possibility for further research on molecular mechanisms.^[10]^

Therefore, in order to explore the role of lncRNAs in the exact pathogenesis of EC, we provide a new basis for understanding the pathogenesis, identify new prognostic indicators, and suggest possible molecular targets for its treatment for patients with EC.

## 2. Materials and methods

### 2.1. Gene expression profiling data

We downloaded the gene expression profiles from the TCGA database. The RNA expression data included 552 cases of EC and 35 normal controls. The miRNA expression data included 546 cases of EC and 33 normal controls. Our study didn’t involve human beings or animals, so the approval of Ethics Committee isn’t necessary in our study.

### 2.2. Screening of differentially expressed genes (DEGs)

After downloading the RNA expression data, we extracted the matrix of Ensembl, transformed it with Homo_sapiens, GRCh38.89.chr.gtf.gz file, and obtained the symbol matrix, including mRNA and lncRNA. We ran the Perl script to extract the matrix of the symbol of the mRNA and lncRNA. After the miRNA-sequencing expression data were downloaded, the matrix of the miRNA was extracted by the Perl script. The corresponding DEGs were obtained by processing and analyzing the mRNA, lncRNA, and miRNA matrices by using the R software, “edgeR” package. Screening conditions |logFold Change|>2, and the false discovery rate <0.01.

### 2.3. Prediction of the interaction between lncRNA and miRNA by the miRcode database

The miRcode (http://www.mircode.org/mircode/) database is a network search platform specially recording and retrieving the interactions between lncRNA and miRNA. It was developed by researchers from Gothenburg University, Sweden. In this software platform, the target of miRNA can be predicted by input-related lncRNA and miRNA. Currently, the latest version of miRcode has completely covered a complete transcriptional group of GENECODE annotations, including 10,419 registered lncRNAs.^[11]^ The miRcode database was used to predict the interaction between miRNA and lncRNA.

### 2.4. Prediction of mRNA targeted by miRNA

miRTarBase is a network database used to retrieve the relationship between miRNA and its mRNA targets. The database provides the latest and comprehensive miRNA-target interaction information, and all miRNA-mRNA interaction data in the database have been verified by at least 2 experiments, including western blotting, real-time fluorescence quantitative polymerase chain reaction, microarray and binding site analysis.^[12]^ MiRDB (http://mirdb.org) is an online miRNA target gene prediction and annotation database. Based on the experimental method of high throughput sequencing, the target gene was predicted by using the bioinformatics tool MirTarget. The miRDB database consists mainly of 5 species of miRNA target genes and related functional annotation information: human, rat, mouse, dog, and chicken. By the 2021 data system update, the miRDB database had included 6709 miRNAs and targeted 21,000 target genes.^[13]^ The TargetScan database was used to predict the target genes by searching for the conservative 8 mer and 7 mer sites that match each of the miRNA seed regions.^[14]^ Three databases were used to predict the miRNAs target mRNAs, and the results were intersected. To further ensure the reliability of the ceRNA regulatory network, we only screened differentially expressed (DE) lncRNA, DEmiRNA, and DEmRNA in tumor tissues and normal tissues to develop the ceRNA network and used Cytoscape 3.7.1 software to visualize the results. The network is the core of cytoscape software, and each node is a gene, protein or molecule, and the connection between nodes represents the interaction of these molecules that can be used to find the interactive relationship among the DEGs in EC.

### 2.5. Gene Ontology (GO) and Kyoto Encyclopedia of Genes and Genomes (KEGG) pathway analysis

The DAVID database is a bioinformatics database, which integrates biological data and analysis tools. The gene function of the candidate can be enriched and analyzed, and the genes are annotated. DAVID online analysis software is used to carry out GO function annotation for DEmRNA in the ceRNA network, with screening conditions of *P* < .05. We used KOBAS 3.0 to perform KEGG analysis of DEmRNA in the ceRNA network.

### 2.6. Survival analysis

The patient’s survival data was extracted from the TCGA database clinical information file. Kaplan–Meier survival curve was carried out to assess the overall survival (OS) difference between the high expression and low expression groups by using log-rank tests. We used the R software “survival” package to perform bilateral pair rank test evaluation of DEmRNA, DElncRNA, and DEmiRNA, and *P* < .05 was considered as significant.

## 3. Results

### 3.1. Identification of DEGs

**Figure 1.**
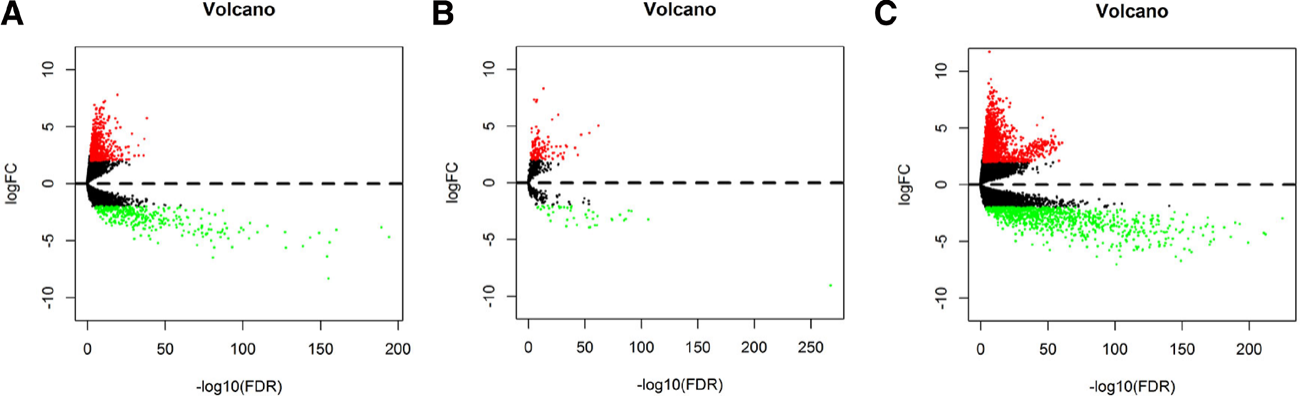
Volcano plot indicating the relation between FDR of the expression of DEGs and fold change in the endometrial cancer group versus the control group. (A) lncRNA; (B) miRNAs; (C) mRNAs. Note: the red spots represent upregulated genes with |Fold Change|>2.0 and FDR < 0.05. The green spots represent downregulated genes with |Fold Change|>2.0 and FDR < 0.01. The black spots represent genes without significant differences. DEGs = differentially expressed genes, FDR = false discovery rate, lncRNA = long non-coding RNA, miRNAs = micro RNAs, mRNAs = messenger RNAs.

**Figure 2.**
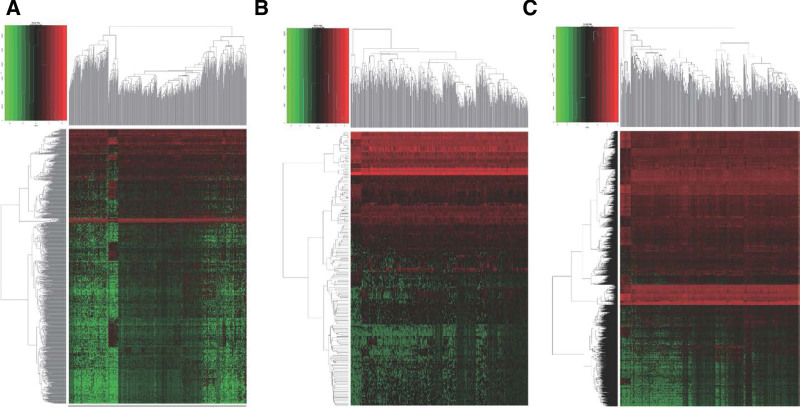
Heat map showing the expression profiles of the (A) lncRNAs, (B) miRNAs and (C) mRNAs. Note: The maps are based on the expression values of all expressed lncRNAs, miRNAs, and mRNAs detected by microarray probes. Each column represents 1 sample, and each row indicates 1 gene. lncRNAs = long non-coding RNAs, miRNAs = micro RNAs, mRNAs = messenger RNAs.

The RNA-sequencing expression profiles downloaded from the TCGA database included 552 EC samples and 35 normal control samples. The total number of DEmRNAs was 2614, including 1644 upregulated and 970 downregulated mRNAs. The total number of DElncRNAs was 1103, with 768 upregulated and 335 downregulated. The total number of DEmiRNAs was 189, of which 140 were upregulated and 49 were downregulated. The DEGs volcano map for the EC sample group and the normal control sample group is shown in Figure 1. Hierarchical clustering heatmap of DGEs are shown in Figure 2.

### 3.2. Construction of ceRNA network in EC

**Figure 3.**
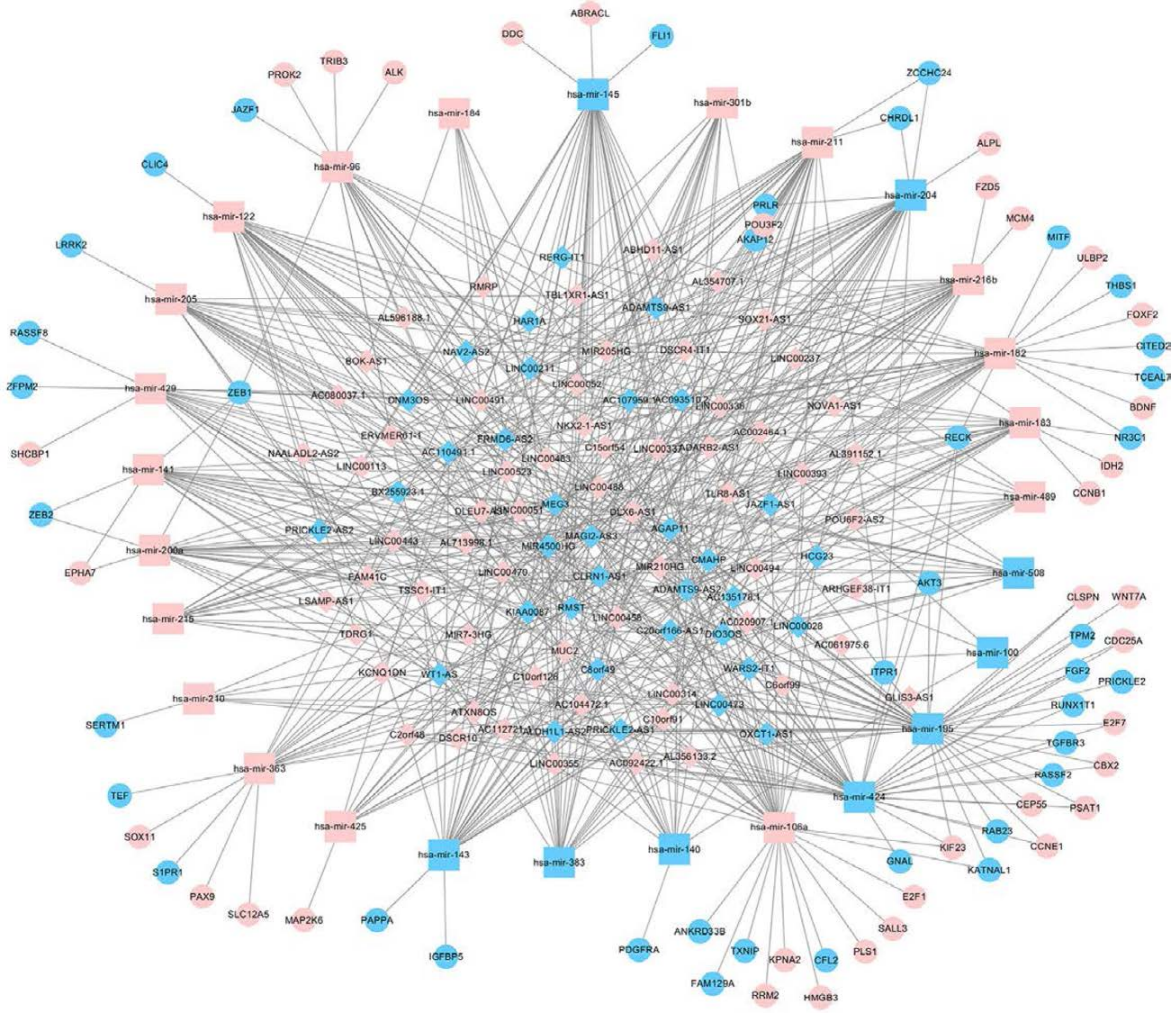
Co-expression ceRNA network of 96 significant lncRNA, 27 significant miRNAs and 74 mRNAs. Diamond: lncRNA; Rectangle: miRNA; Ellipse: mRNA. The blues represent downregulated genes. The pinks represent upregulated genes. ceRNA = competing endogenous RNAs, lncRNA = long non-coding RNA, miRNAs = micro RNAs, mRNAs = messenger RNAs.

To further investigate the role of DElncRNA in EC and its interaction between lncRNA and miRNA, we further constructed a ceRNA network of lncRNA-miRNA-mRNA expression. By using the miRcode database to perform lncRNA and miRNA comparisons, 1103 differences in lncRNA were compared with 189 DEmiRNA, among which 96 DElncRNA and 27 DEmiRNA interactions were obtained. We predicted the targets of 27 DEmiRNA by using miRTarBase, miRDB and the TargetScan database, and the predicted target genes were intersected. A total of 2540 target genes were predicted by the 3 databases. The predicted target genes were intersected with 2614 DEGsmRNA to get 74 DEmRNA. As shown in Figure 3, 96 DElncRNAs, 27 DEmiRNAs, and 74 DEmRNAs were involved in the construction of the ceRNA regulatory network.

### 3.3. GO and KEGG pathway analysis

**Figure 4.**
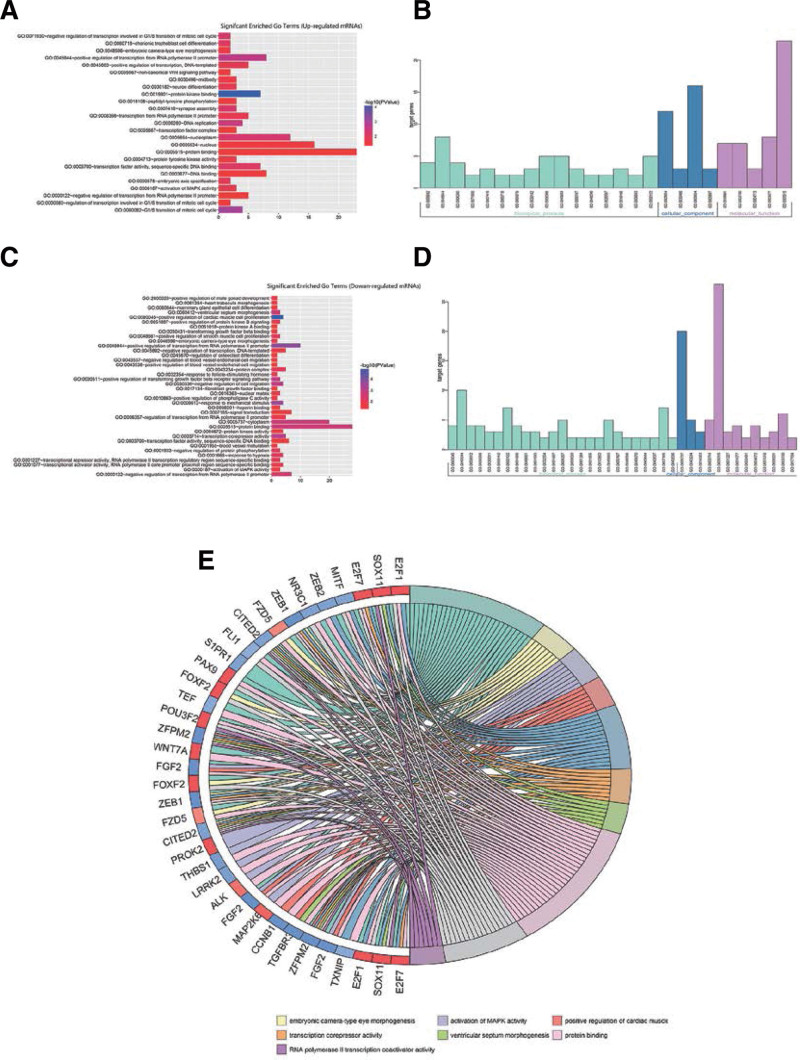
GO analysis of DEmRNA in EC. (A and B) GO annotation of upregulated mRNAs including biological processes, cellular components and molecular functions. (C and D) GO annotation of downregulated mRNAs including biological processes, cellular components and molecular functions. (E) Distribution of DEmRNA in GO terms. DEmRNA = differentially expressed messenger RNA, EC = endometrial cancer, GO = gene ontology, mRNAs = messenger RNAs.

GO analysis of DEmRNAs can indirectly reflect the biological function of lncRNAs and miRNAs. The results indicated that in biological process, the high-expressed genes were enriched in mitotic cell cycle and cell differentiation (Fig. 4A and B), and the low-expressed genes were enriched in cell proliferation, cell migration, response to mechanical stimulus and hypoxia, and signal transduction (Fig. 4C and D). In the molecular function, the upregulated genes were mainly involved in protein kinase binding, transcription factor activity and deoxyribonucleic acid binding, and the downregulated genes were mainly involved in protein binding, transcription factor activity and growth factor binding. In terms of cell composition, the upregulated genes were mainly involved in the nucleoplasm, midbody and nucleus, and the downregulated genes were mainly involved in the formation of the cytoplasm, protein complex and nuclear matrix. Distribution of GO term showed that these DEmRNAs were enriched in activation of mitogen-activated protein kinase activity, positive regulation of cardiac muscle, and protein binding (Fig. 4E).

**Figure 5.**
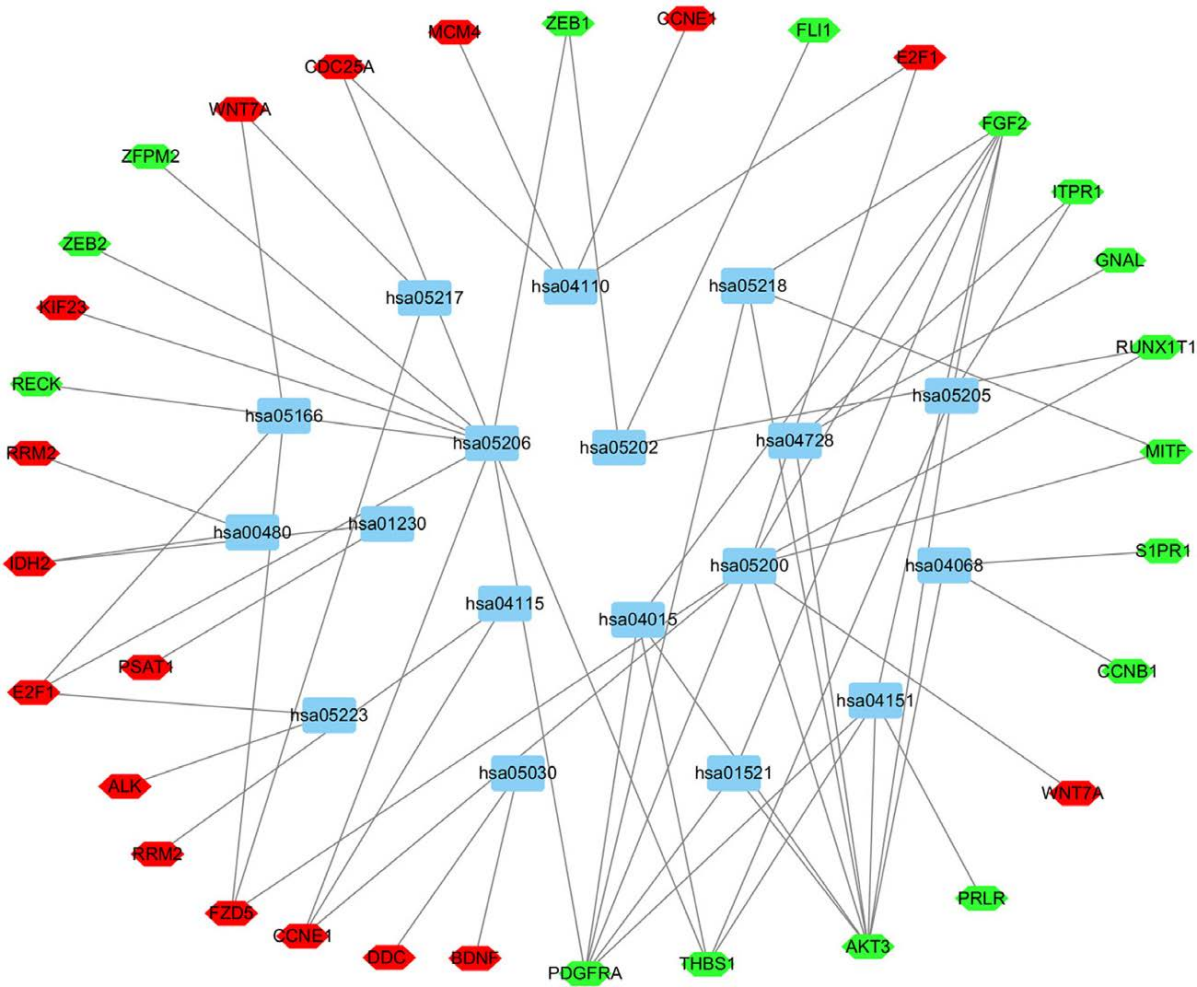
Significant pathway enrichment analysis of DEGs. The blue represents the signal pathway, the green represents the downregulated genes and the red represents the upregulated genes. DEGs = differentially expressed genes.

We identified the upregulation of 10 pathways related to upregulated DEmRNAs and 10 pathways related to downregulated DEmRNAs. The upregulated DEmRNA pathway was mainly enriched in the cell cycle, microRNAs in cancer and pathways in cancer, and the p53 signaling pathway. The result of our study suggested that these pathways may produce a marked effect in the occurrence and development of EC. The pathway and gene information were imported into the Cytoscape to visualize the results (Fig. 5).

### 3.4. Survival and Cox regression analysis

**Figure 6.**
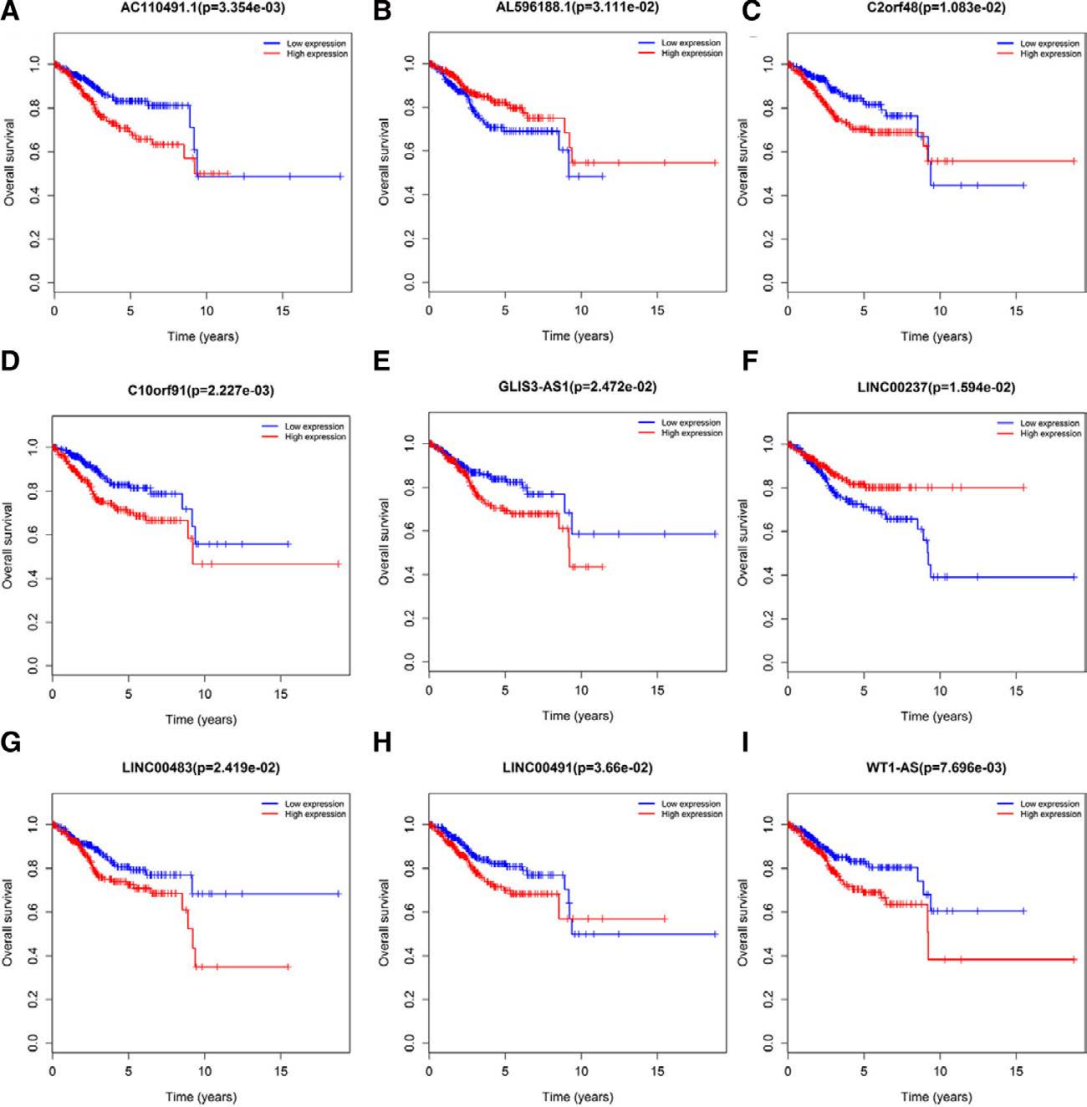
Kaplan–Meier curve analysis of DElncRNAs and overall survival rate in patients with EC. There were 9 DElncRNAs (*P* < .05), including (A) AL596188.1, (B) LINC00237, (C) C10orf91, (D) GLIS3-AS1, (E) LINC00483, (F) WT1-AS, (G) C2orf48, (H) LINC00491, and (I) AC110491.1. DElncRNAs = differentially expressed long non-coding RNAs, EC = endometrial cancer.

**Figure 7.**
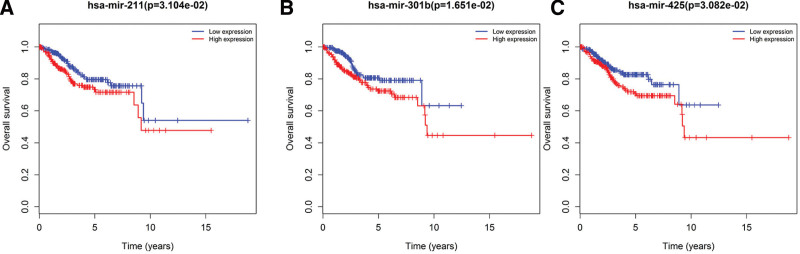
Kaplan–Meier curve analysis of DEmiRNA, and overall survival rate in patients with EC. There were 3 DEmiRNAs (*P* < .05), including (A) hsa-mir-301b, (B) hsa-mir-211, and (C) hsa-mir-425. DEmiRNA = differentially expressed micro RNA, EC = endometrial cancer.

**Figure 8.**
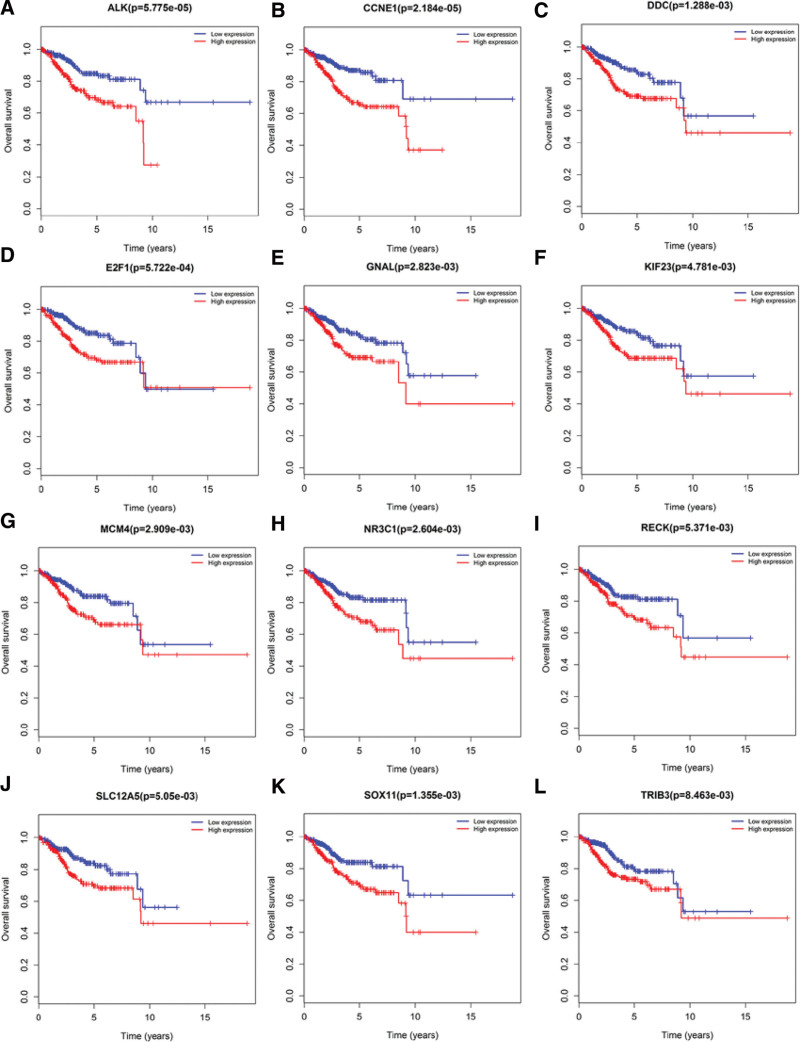
Kaplan–Meier curve analysis of DEmRNA and overall survival rate in patients with EC; 12 DEmRNAs are shown (*P* < .05), including (A) ALK, (B) CCNE, (C) DDC, (D) E2F1, (E) GNAL, (F) KIF23, (G) MCM4, (H) NR3C1, (I) RECK, (J) SLC12A5, (K) SOX11, and (L) TRIB3. DEmRNA = differentially expressed messenger RNA, EC = endometrial cancer.

In this study, we used Kaplan–Meier curve analysis to investigate whether these 96 DElncRNA, 27 DEmiRNA, and 74 DEmRNA in EC patients were correlated with OS rates. The results of survival analysis revealed that a total of 9 DElncRNAs were significantly related to the OS rate of the patients. The expression of AL596188.1 and LINC00237 was positively correlated with OS. The expressions of C10orf91, GLIS3-AS1, LINC00483 and WT1-AS were negatively correlated with OS. Two DElncRNAs (C2orf48 and LINC00491) were negatively correlated with the patient’s 10-year survival rate, and the survival rate >10 years was also positively correlated. The expression of AC110491.1 was negatively correlated with the patient’s 10-year survival rate (Fig. 6). Among the 27 miRNAs that participated in the construction of the ceRNA network, 3 DEmiRNAs (hsa-mir-301b, hsa-mir-211 and hsa-mir-425) were significantly correlated with the OS rate of the patients (Fig. 7). There were 74 mRNAs in the ceRNA network construction. The Kaplan–Meier curve analysis showed that there were 12 DEmRNAs (ALK, CCNE, DDC, E2F1, GNAL, KIF23, MCM4, NR3C1, RECK, SLC12A5, SOX11, and TRIB3) that were significantly correlated with the OS rate (*P* < .05) (Fig. 8).

## 4. Discussion

EC causes significant mortalities worldwide in the clinic. Although surgery and radiotherapy can relieve symptoms in most patients, the prognosis of advanced EC is relatively poor, and the 5-year survival rate of patients is relatively low, which is a serious threat to the health and safety of women.^[15]^ With the in-depth study of lncRNA recently, a great number of lncRNAs have been reported to be involved in the occurrence, development, metastasis and recurrence of EC.^[16]^ Recent studies have indicated that lncRNA work as a ceRNA to combine with miRNA, participating in the expression and function of target gene, which may play a significant role in genesis and development of cancers. Several ceRNA regulatory networks have been developed to improve prognostication of cancers and have been verified by experiments. BGL3 competes with PTEN to combine with miR-17, miR-93, miR-20a, miR-20b, miR-106a, and miR-106b, affecting the PTEN gene expression level and downstream signaling pathways, thereby affecting tumor growth.^[17]^ As a ceRNA, HULC and miR-372 competitively combine with PRKACB in hepatocellular carcinoma, thereby reducing the transcriptional inhibition effect of miR-372 on target genes and upregulating PRKACB expression, thereby promoting the CREB phosphorylation level.^[18]^ However, there is still a lack of comprehensive analysis of lncRNA- and miRNA-related ceRNA in EC in the whole genome range, particularly based on high-throughput detection and a large sample size of EC.

In this study, we used the R software package to analyze the downloaded expression data and identified 2614 DEmRNAs, 1103 DElncRNAs, and 189 DEmiRNAs. Through miRcode, miRTarBase, miRDB, and TargetScan database prediction, further studies found that 96 DElncRNAs, 27 DEmiRNAs, and 74 DEmRNAs participated in the construction of a ceRNA regulatory network. At present, many protein coding genes have been reported to be involved in the development of EC in our ceRNA network. TCEAL7, can inhibit the proliferation and invasion of EC cells as a tumor suppressor gene.^[19]^ The high level of FGF2 in EC patients is an independent risk factor and is closely related to the poor prognosis of EC.^[20]^ The expression level of ZEB1 is closely related to tumor typing, classification, myometrium infiltration and lymph node metastasis. Therefore, ZEB1 can be used to predict the risk of lymph node metastasis by its preoperative characteristics. The specificity was 96.2%, and the sensitivity was 62.1%.^[20]^ In total, in view of their important role, they are expected to be new early diagnostic, prognostic and therapeutic targets for EC.

lncRNA is a competitive ceRNA combining with miRNA, which regulates the protein level of the encoding gene and participates in the regulation of the biological behavior of the cells. Therefore, the functional analysis of mRNA can be used to predict the biological functions of lncRNA and miRNA indirectly. The GO annotation results revealed that in terms of cell composition, the high-expressed genes are mainly enriched in nucleoplasm, midbody and nucleus, and the low-expressed genes participated in the formation of cytoplasm, protein complex and nuclear matrix. Further analysis by KEGG pathway enrichment revealed that the upregulated DEmRNA pathway was mainly concentrated in cell cycle, microRNAs in cancer and pathways in cancer, and p53 signaling pathways. The pathways which were enriched by low expression of DEmRNA were mainly concentrated in microRNAs in cancer, PI3K-Akt signaling pathway, Rap1 signaling pathway, and EGFR tyrosine kinase inhibitor resistance. A previous study found that the activation of PI3K-Akt and P53 signaling pathways had a very high risk of EC progression.^[21]^ In the Rap1 signaling pathway, Rap1GAP can act as a tumor suppressor inhibiting the Ras superfamily protein Rap1 in EC, and it is an independent prognostic factor in EC.^[22]^ EGFR tyrosine kinase inhibitor is a small molecule EGFR inhibitor that can inhibit tyrosine kinase activation, block the EGFR signaling pathway, inhibit the proliferation and metastasis of tumor cells, and promote apoptosis through competitive endogenous ligand binding to EFGR.^[23]^ Therefore, lncRNA may regulate the occurrence and development of tumors by regulating the expression of target genes by participating in the functional and signaling pathways listed above.

In general, we conducted survival curve analysis to evaluate correlation between DElncRNA, DEmiRNA, and DEmRNA expression levels and the OS time of EC patients. The patients with EC were classified into a low expression and a high expression group. The results showed that a total of 9 DElncRNAs were significantly associated with the OS rate of the patients. These lncRNAs have been identified for the first time in EC and may be closely related to patient outcomes. In future clinical practice, they may provide insights into the targeted therapy of EC.

Our results showed that 3 DEmiRNAs (hsa-mir-301b, hsa-mir-211, and hsa-mir-425) were negatively correlated with the OS rate of the patients. It has been shown that overexpression of hsa-mir-301b can promote the proliferation, migration, and epithelial mesenchymal transition signals of tumor cells.^[24]^ The results of that study are in accordance with our findings. The role of hsa-mir-211 in tumors is controversial. A study reported that hsa-mir-211 can promote the proliferation and invasion of lung cancer cell lines by targeting MxA.^[25]^ However, another study found that hsa-mir-211 can inhibit cervical cancer cell invasion and epithelial-mesenchymal transition by targeting MUC4.^[26]^ At present, there is no research report on the role of hsa-mir-211 in EC, and further experimental research is needed. One study reported that micoRNA-425-5p is a potential prognostic biomarker for EC and has potential therapeutic value in EC.^[27]^ Additionally, microRNA-425-5p can promote the invasion and migration of hepatoma cells by regulating a variety of signaling pathways.^[28]^ This is consistent with our findings, suggesting that micoRNA-425-5p may play an important role in the development of EC and is expected to become a new target for the diagnosis and treatment of EC.

Our study found that 12 DEmRNAs (ALK, CCNE, DDC, E2F1, GNAL, KIF23, MCM4, NR3C1, RECK, SLC12A5, SOX11, and TRIB3) were significantly correlated with the OS rate of patients. It has been reported that ALK1 can activate transforming growth factor-β signaling, which is necessary for angiogenesis during tumor growth. The expression of ALK1 in EC is significantly elevated and promotes the growth of EC cells.^[29]^ CCNE was a prognostic factor for EC.^[30]^ miR-145 can inhibit EC cell growth by inhibiting the expression of SOX11.^[31]^ In addition, we found that in the ceRNA regulatory network, has-mir-363 can also regulate the expression of SOX11. The other mRNAs have not been previously reported in EC, and their roles still need to be verified by experiments.

miRNA, as a research hotspot in cancer, has made rich research achievements in many kinds of tumors, such as EC. We found that in the ceRNA regulatory network, 1 miRNA can regulate multiple target genes, and a single target gene may be regulated by multiple miRNAs. Therefore, there are some problems in the treatment of a single miRNA target; that is, any changes in miRNA can lead to the change in gene expression of multiple non-target genes. On the other hand, a change in the expression of a miRNA is not sufficient for target gene treatment. In addition, multiple lncRNAs can regulate the expression of a target gene by binding miRNA competitively. It has been suggested that multiple lncRNAs could act as ceRNA to adsorb 1 miRNA, which can effectively change the expression of the target genes. Disorder of the ceRNA network can lead to many cancers.^[32]^ Therefore, the specific mechanisms of lncRNA and miRNA in cancer, especially in EC, still need further exploration and research to provide a basis for targeted therapy in EC.

In conclusion, we downloaded the expression profiles from the TCGA database. After screening, we obtained 96 DElncRNAs, 27 DEmiRNAs, and 74 DEmRNAs that participated in the construction of a ceRNA regulatory network. The GO and KEGG signaling pathways of mRNA were used to indirectly detect the biological function and signaling pathways regulated by lncRNAs. It was also found that 9 DElncRNAs, 3 DEmiRNAs, and 12 DEmRNAs were closely related to the OS of patients with EC. Our study provides a new insight into understanding the cause and underlying molecular events in EC. The ceRNA-mediated gene regulation network provides new molecular targets for the diagnosis, treatment and prognostication of EC. However, further molecular biological experiments are required to confirm the function of the identified genes in EC.

## Author contributions

**Conceptualization:** Fengfan Li.

**Data curation:** Chunlei Zhou.

**Formal analysis:** Chunlei Zhou.

**Funding acquisition:** Shuxuan Li.

**Investigation:** Chunlei Zhou, Shuxuan Li.

**Methodology:** Jingyu Wang.

**Project administration:** Hong Mu.

**Software:** Fengfan Li.

**Supervision:** Shuxuan Li.

**Validation:** Chunlei Zhou, Jingyu Wang, Hong Mu.

**Writing – original draft:** Fengfan Li.

**Writing – review & editing:** Fengfan Li, Hong Mu.
